# Fluorine-Free Membranes Consisting of a Blend of S-PVA and PEBAX 1657 for Proton Exchange Membrane Fuel Cells: The Role of Titanium Dioxide Phosphate (TiO_2_PO_4_) Nanoparticle Fillers

**DOI:** 10.3390/membranes15090280

**Published:** 2025-09-18

**Authors:** Manhal H. Ibrahim Al-Mashhadani, Gábor Pál Szijjártó, Asmaa Selim, Zoltán Sebestyén, Judith Mihály, András Tompos

**Affiliations:** 1 Institute of Materials and Environmental Chemistry, HUN-REN Research Centre for Natural Sciences, Magyar Tudósok Körútja 2, H-1117 Budapest, Hungary; manhal.ibrahim@ilps.uobaghdad.edu.iq (M.H.I.A.-M.); szijjarto.gabor@ttk.hu (G.P.S.); asmaa.selim@ttk.hu (A.S.); sebestyen.zoltan@ttk.hu (Z.S.); mihaly.judith@ttk.hu (J.M.); 2 Hevesy György Doctoral School of Chemistry, Eötvös Loránd University, Pázmány Péter Sétány 1/A, H-1117 Budapest, Hungary; 3 Institute of Laser for Postgraduate Studies, University of Baghdad, Baghdad 10070, Iraq; 4 Chemical Engineering and Pilot Plat Department, Engineering and Renewable Energy Research Institute, National Research Centre, 33 El Bohouth Street, Giza 12622, Egypt

**Keywords:** proton exchange membrane, fuel cell, fluorine-free, sulfonated polyvinyl alcohol (S-PVA), PEBAX, titanium dioxide phosphate (TiO_2_PO_4_)

## Abstract

Novel blend membranes containing S-PVA and PEBAX 1657 at a blend ratio of 8:2 were doped with varying amounts of titanium dioxide phosphate (TiO_2_PO_4_) as a nanoparticle filler at concentrations of 0, 3, 5, and 7 wt%. The membranes were fabricated using the solution-casting technique. The effect of the TiO_2_PO_4_ nanofiller on the polymer matrix was thoroughly investigated. Our aim was to investigate how the incorporation of TiO_2_PO_4_ nanofillers into non-fluorinated SPP-based membranes affects their structural, physicochemical, and electrochemical properties for application in fuel cells. Crystallinity of the samples was checked by means of X-ray diffraction (XRD), while FTIR was used to investigate the contact between the nanofiller and the polymers. The good compatibility resulted in strong interactions between the constituents and led to increased crystallinity of the membrane as well. Furthermore, SEM images confirmed the uniform distribution of the nanofiller. These structural features led to good thermal stability, as evidenced by thermogravimetric analysis (TGA), and good mechanical strength, as proved by tensile tests. Among the samples investigated, the highest water uptake of 51.70% was achieved on the composite membrane containing 3 wt% TiO_2_PO_4_, which also showed the highest ion exchange capacity at room temperature, reaching 1.13 meq/g. In line with these properties, among the synthesized membranes, the membrane labeled SPP 3% TiO_2_PO_4_ has the highest current density and power density, with values of 175.5 mA/cm^2^ and 61.52 mW/cm^2^, respectively.

## 1. Introduction

The need for energy and people’s dependence on it are escalating in direct proportion to both technological advancements and population growth rates. Fuel cells are a promising technology for energy conversion that can meet this increasing energy demand [1].

Fuel cells generally come in several types, including solid oxide fuel cells (SOFCs), alkaline fuel cells (AFCs), phosphoric acid fuel cells (PAFCs), molten carbonate fuel cells (MCFCs), and polymer electrolyte membrane fuel cells (PEMFCs). Each is distinguished by unique characteristics and operational limitations; for example, SOFCs and MCFCs operate at high temperatures, whereas AFCs and PAFCs rely on particular electrolytes that limit their use on a large scale. Conversely, PEMFCs have attracted a lot of interest because of their low-temperature operation, fast start-up, high power density, and compactness, which make them exceptionally well-suited for transportation, portable electronics, and stationary power systems [2]. Proton exchange membrane fuel cells (PEMFCs) are gaining increased interest due to their advantageous operational conditions, extensive applications, and high efficiencies [3]. Often described as the “heart” of a PEM (proton exchange membrane) fuel cell, the proton exchange membrane (PEM) is crucial for the cell’s functionality [4]. PEMFCs necessitate membranes with specific qualities such as superior proton conductivity, minimal fuel crossover, thermal and chemical durability, robust mechanical strength, and affordability due to their exceptional proton conductivity and notable mechanical strength [5]. PEM fuel cells use polyperfluorosulfonic acid (PFSA) membranes, including Nafion, as electrolyte membranes due to their exceptional proton conductivity, robust mechanical strength, and superior chemical and thermal stability [6]. Nevertheless, the substantial cost of Nafion and its reduced stability at elevated temperatures hinder its widespread use [7,8]. Compared to non-fluorinated membranes, there are some merits to them, including lower cost, easy synthesis, less environmental impact, and tunability of functionalization to enable higher proton conduction. Non-fluorinated membranes with inorganic fillers can also augment mechanical stability, thermal robustness, and electrochemical performance to become alternative competitors in sustainable and commercially viable fuel cell applications. There are practical and potential concerns during the operation of fuel cells; oxygen that enters the three-phase interface, where the PEM, catalyst, and GDL meet, can react with protons that have been conducted to the cathode through the PEM from the anode to produce hydrogen peroxide (H_2_O_2_) as a side reaction. H_2_O_2_, under the catalytic action of certain metal ions, generates •OH• and HOO• radicals, which will selectively attack the weak parts of the membrane, such as C-O-C and the H-containing terminal end groups (-CF_2_COOH), causing its irreversible chemical degradation, thereby reducing its proton conductivity and the service life of the fuel cell. When radicals attack the C-F bonds in the side chains, causing them to break, H and F can also produce HF, a strong acid, which accelerates the degradation of the catalyst, reducing its loading, activity, and membrane, ultimately affecting its performance through a reduction in ion exchange capacity (IEC), a loss of proton conductivity, and thinning. This has made the development of a fluorine-free PEM with excellent mechanical, chemical, and thermal stability a popular research direction [9]. Suitable alternatives are needed, particularly when operating at elevated temperatures or reduced humidity [10]. One strategy to enhance the performance of the proton exchange membrane is to blend different polymers [11]. Membranes were developed using a range of hydrocarbon polymers, such as polyether ketone (PEEK) [12], polyarylene ether sulfone [13], polybenzimidazole (PBI) [14], polyether sulfone (PES) [15], polyamide [16], and polyvinyl alcohol (PVA) [17].

PVA stands out among these polymers due to its high hydrophilicity, biodegradable chemical structure, ease of modification, and superior film-forming capabilities [18,19,20]. However, it has several limitations, such as being water soluble, having low strength, low proton exchange capacity, and poor thermal stability, which prevent it from meeting all the required membrane performance characteristics on its own [21,22,23]. To improve the proton conductivity of PVA, it is proposed to modify it with sulfonic acid groups. Modification with sulfosuccinic acid (SSA) not only improves proton conductivity but also the mechanical stability of PVA membranes by acting as a crosslinking agent. Several studies have examined the properties of crosslinked PVA/SSA membranes as a function of SSA content [24,25]. These studies have confirmed the viability of these crosslinked polymers in fuel cell applications [26].

Poly (ether–block–amide) polymers, available commercially as PEBAX^®^, are widely utilized due to advantageous properties such as high permeability, selectivity, and durability [27]. The crystalline structure of the polyamide segments determines the mechanical stability of these copolymers. On the other hand, the amorphous polyether segments enable the efficient transport of organic materials [28]. These polymers are made up of rigid polyamide and flexible polyether segments that are chemically bonded together to make a thermoplastic copolymer [29].

PEBAX copolymers are synthesized by copolymerizing various poly (amides), including PA6, PA12, and PA66, with different poly (ethers), like polyethylene oxide (PEO) and poly (tetramethylene oxide) [30]. PEBAX 1657 is composed of 40% amide and 60% ether segments. The amide segment (PA6) acts as the hard section, offering hydrophilic sites for ionic exchanges, while the ether segments (PEO) act as the soft section, enhancing mass transfer. PEBAX 1657 has a unique crystalline-amorphous structure that combines the best features of thermoplastics and elastomers. This makes it a material with high chemical resistance and mechanical strength [31].

The prevailing trend in developing new polymeric membranes for fuel cells is the addition of doping agents into the polymer matrix [32]. Metal oxides, like ZrO_2_, SiO_2_, and TiO_2_, known for their easy customization and accessibility, are extensively utilized as additives to create nanocomposite membranes. These membranes exhibit improved characteristics, such as enhanced chemical and mechanical stability, reduced fuel permeability, and increased ionic conductivity, all of which collectively improve fuel cell performance [33]. TiO_2_, an inorganic material, is noted for its adaptable properties, which often enhance the mechanical strength, thermal stability, non-flammability, and corrosion resistance of polymeric composites [34,35]. Additionally, it is cost-effective. The high surface area of modified TiO_2_ nanoparticles, used as a doping agent in polymer electrolytes, significantly boosts the performance of fuel cells and batteries [35]. Functionalization of TiO_2_ makes the polymer matrix and TiO_2_ particles more compatible and creates interconnecting ion channels that improve ion transport [36]. Treating TiO_2_ with phosphoric acid to bind phosphate anions introduces acidic groups to the material, which is anticipated to improve its ionic conductivity, water adsorption, and ion exchange capacity while also reducing fuel permeation through the membranes. The integration of nanosized TiO_2_ powders into various polymer matrices has been extensively studied [37].

In our multicomponent system consisting of PVA, PEBAX, and phosphated titania, we aim to understand the working mechanism of proton conduction and the contribution of different constituents to it in order to improve performance for practical use. The use of PEBAX in polymer blends for fuel cell applications has been scarcely investigated [27]. In our previous work, blends of PEBAX 1657 and sulfonated S-PVA were modified with sulfated montmorillonite [38]. The current study examines how different amounts of TiO_2_PO_4_ filler (0, 3, 5, and 7 wt%) affect the water uptake, swelling degree, thermal stability, chemical stability, ion exchange capacity, and fuel cell efficiency of membrane blends consisting of PEBAX 1657 and S-PVA. The hydrophilic and hydrophobic segments of PEBAX 1657 should provide morphological and mechanical stability as well as result in the formation of proton conduction channels due to phase separation. S-PVA prepared with SSA as both a crosslinking and sulfonating agent improves membrane hydrophilicity and reduces the swelling of PVA.

## 2. Experimental

### 2.1. Materials

Arkema in Colombes, France, delivered PEBAX 1657, which comprises 60 wt% polyether oxide (PEO) and 40 wt% polyamide (PA6). High-molecular-weight PVA (Mw = 85,000–124,000 g/mol) was purchased from Sigma-Aldrich (St. Louis, MO, USA), which has a hydrolysis level of over 99%. Solution of SSA (70% in water) from Sigma-Aldrich (USA) was used as received without further purification. Titanium (IV) oxide rutile powder (TiO_2_, <5 µm, ≥99.9 wt% from Sigma-Aldrich), H_3_PO_4_ (85 wt% from Fisher Chemical), and H_2_SO_4_ (98 wt% from Spectrum Chemical MFG Corp.) were employed to synthesize titanium oxide phosphate (TiO_2_PO_4_). We obtained a 5 wt% copolymer resin solution of DuPont Nafion (D520–1000 EW) from the Fuel Cell Store. Solvents, such as ethanol and 2-propanol, are delivered from Gilca, Lab Box, and Schar Lab, Barcelona, Spain. We used Millipore water for all aqueous solution preparations.

### 2.2. Synthesis of Titanium Dioxide Phosphate (TiO_2_PO_4_) Nanoparticles

The functionalization of TiO_2_ with the PO_4_^−3^ anion was achieved through an impregnation–calcination technique. The process to obtain TiO_2_PO_4_ powder consisted of the following steps. In a round-bottom flask immersed in a water bath, TiO_2_ powder was suspended in a 0.1 M aqueous solution of H_3_PO_4_ in a 1:1 molar ratio of TiO_2_ to H_3_PO_4_, and the mixture was stirred at 80 °C for 1 h. The mixture was then filtered and dried in an air oven at 110 °C for one day. Subsequently, the material was calcined at 450 °C in a muffle furnace.

### 2.3. Preparation of Membranes

**Figure 1:**
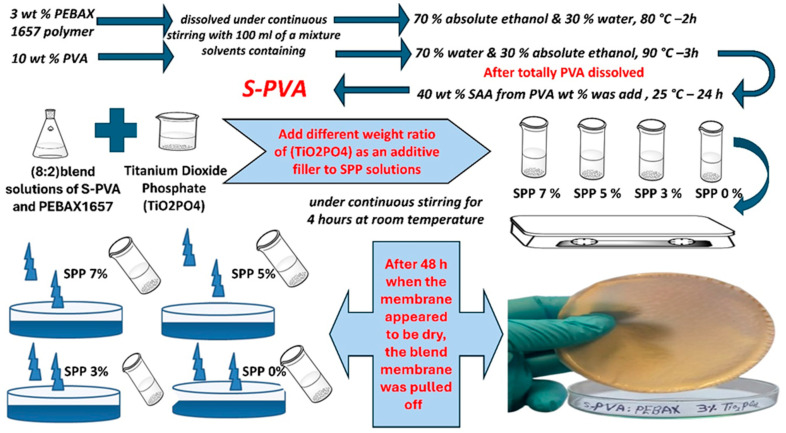
The main steps of the fabrication procedure of blend membranes with different titanium dioxide phosphate (TiO_2_PO_4_) content.

A 3 *w*/*v*% solution of PEBAX 1657 was prepared by dissolving it in a 7:3 volume ratio solution of absolute ethanol and water under reflux for 2 h at 80 °C. Similarly, 10 *w*/*v*% PVA was prepared by dissolving it in a mixture of water and absolute ethanol (7:3 *v/v* ratio) under stirring and reflux for 3 h at 90 °C. After the PVA was completely dissolved and the solution cooled, the SSA solution was added. This mixture was then continuously stirred at room temperature for 24 h. Nominal SSA content of the SSA-PVA membrane was 40 wt%. Different amounts of titanium dioxide phosphate (TiO_2_PO_4_) (3, 5, and 7 wt%) were subsequently added to a solution of S-PVA and PEBAX 1657 at a blend ratio of 8:2 and stirred for 4 h at room temperature. The resulting solutions were cast onto glass Petri dishes and allowed to dry for 48 h. Figure 1 shows the main steps of the fabrication procedure of membranes.

For ease of reference, Table 1 defines the designation of membranes prepared with different amounts of titanium dioxide phosphate. SPP refers to sulfonated polyvinyl alcohol and poly (ether–block–amide).

### 2.4. Membrane Characterization

For each measurement, three values were taken, which were then averaged to increase statistical accuracy.

#### 2.4.1. Water Uptake (WU) and Swelling Ratio (SR)

To assess the water uptake (WU) and swelling ratio (SR) of the membranes, 2 × 2 cm^2^ square samples were soaked in deionized (DI) water for 24 h at 80 °C. After removing the sample from the bath, the surface water was removed with a tissue, and the weight and dimensions of the wet membranes were measured. The samples were then dried overnight in a vacuum oven at 50 °C. After drying, the mass and dimensions of the samples were recorded again. Calculations for water uptake and in-plane (area) and through-plane (thickness) swelling ratios were performed according to Equations (1)–(3), respectively.

(1) $$WU\left(\%\right)=\frac{\left(W_{\mathrm{wet}}-W_{\mathrm{dry}}\right)}{W_{\mathrm{dry}}}\times 100$$

where *W_wet_* and *W_dry_* denote the wet and dry weights of the samples, respectively.

(2) $$SR\;(in-plane\;\%)=\frac{\left(A_{\mathrm{wet}}-A_{\mathrm{dry}}\right)}{A_{\mathrm{dry}}}\times 100$$

The terms “*A_dry_*” and “*A_wet_*” refer to the membrane surface areas in a dry and wet state, respectively.

(3) $$\mathrm{SR}\;(\mathrm{through}-\mathrm{plane}\;\%)=\frac{\left(T_{\mathrm{wet}}-T_{\mathrm{dry}}\right)}{T_{\mathrm{dry}}}\times 100$$

The terms “*T_dry_*” and “*T_wet_*” refer to the membrane thickness in a dry and wet state, respectively [39].

#### 2.4.2. Ion Exchange Capacity (IEC)

The ion exchange capacity (IEC) of the membranes was determined using acid-base titration and expressed in millimoles per gram (meq/g). First, the samples were immersed in 20 mL of 1 M NaCl solution for 24 h, allowing for the substitution of H^+^ ions with Na^+^ ions. Afterwards, the solution, which now contained the released H^+^ ions, was titrated using a 0.1 M NaOH solution. The IEC values were calculated using Equation (4).

(4) $$IEC\;(meq/g)=\frac{\left(C_{\mathrm{NaOH}}\times V_{\mathrm{NaOH}}\right)}{W_{\mathrm{dry}}}$$

where *C_NaOH_*, *V_NaOH_*, and *W_dry_* stand for the concentration of the NaOH titration solution, the volume of NaOH utilized, and the dry weight of the membranes, respectively.

#### 2.4.3. TGA Analysis

The thermal stability of the membranes was assessed by TG analysis using a Perkin Elmer TGS-2 (Eurotherm, UK) thermobalance equipped with a modern furnace and a Eurotherm temperature controller from Worthing, UK. Each sample, approximately 4.5 milligrams in weight, was precisely weighed and placed in a platinum holder under a flow of argon gas at 140 mL/min. The samples were subjected to a heating rate of 20 °C/min, starting from room temperature up to 900 °C.

#### 2.4.4. Scanning Electron Microscopy (SEM)

The surface texture of the composite membranes was investigated using a JEOL JSM-6380 LA (JEOL Ltd., Tokyo, Japan) variable pressure scanning electron microscope (VP-SEM) through scanning electron microscopy (SEM). This microscope is particularly effective for conducting microstructural analyses in high-vacuum environments.

#### 2.4.5. Fourier Transform Infrared Spectroscopy (FTIR)

The interaction between TiO_2_PO_4_ and the membrane composed of S-PVA and PEBAX1657 blend was analyzed using Fourier Transform Infrared Spectroscopy (FTIR) (Varian Inc., Palo Alto, CA, USA) with the attenuated total reflectance (ATR) mode. This study was conducted using a Varian 2000 (Scimitar Series) spectrometer (Varian Inc., Palo Alto, CA, USA), which covers a spectral range of 4000 to 600 cm^−1^. For each membrane, the FTIR spectrum was obtained by averaging 64 scans with a spectral resolution of 4 cm^−1^.

#### 2.4.6. X-Ray Diffraction (XRD)

The X-ray diffraction (XRD) patterns were recorded using a Philips PW 3710 goniometer (Malvern Panalytical Ltd., Prague, Czech Republic) equipped with a PW1050 Bragg-Brentano parafocusing system from Malvern Panalytical Ltd., Prague, Czech Republic. The X-ray source utilized Cu Kα radiation with a wavelength (λ) of 0.15418 nm. Diffraction angles (2θ) were measured across a range from 4° to 75°. The lattice parameters were determined using the Pawley fitting method, which involves a detailed profile fitting approach.

#### 2.4.7. Mechanical Stability

The mechanical properties of all membranes were assessed using a universal testing machine (Zwick Z005 GmbH & Co., KG, Ulm, Germany). Each membrane, with dimensions of 75 mm by 10 mm, was tested at a speed of 20 mm/min with an initial grip distance of 35 mm.

#### 2.4.8. Chemical Stability

The chemical durability of the composite membranes was tested using Fenton’s reagent, consisting of 3 wt% hydrogen peroxide (H_2_O_2_) and 4 ppm of ferrous sulfate (Fe_2_SO_4_), and 2 × 2 cm^2^ square samples were cut out. The samples were then dried in an oven at 60 °C for 24 h. After drying, the samples were accurately weighed using a high-precision balance. They were immersed in Fenton’s solution and kept at 80 °C for 24 h. The samples were then removed, dried, and weighed to calculate weight loss [40].

#### 2.4.9. Electrochemical Characterization and MEA Performance

Fuel cell tests were carried out on a Scribner 850 Fuel Cell Test System. The active area of the MEA is 16 cm^2^, with a loading of 0.15 mgPt/cm^2^ on both the cathode and anode sides. QuinTech C-40-PT was used as a reference catalyst, with 40 m/m% Pt content. Other components of the catalyst ink were Quintech NS05 Nafion solution (5 m/m%) and 2-propanol (99.99 *v*/*v*%, Molar Chemicals, Halásztelek, Hungary). The catalyst ink was applied to the surface of GDL (Freudenberg FCCT SE & Co. KG carbon paper H23C6) by the spray coating method using a Conrad Electronic SE AB200 airbrush. Composite membranes manufactured according to the method detailed above were used in a size of 70 × 70 mm. The gaskets were made of MDPE foil (BRALEN FA 03-01 type from Mizse-Plast company, Lajosmizse, Hungary). The size of the gaskets was 70 × 70 mm with a 41 × 41 mm window and 150-micron thickness.

## 3. Results and Discussion

### 3.1. Water Uptake Capacity and Swelling Ratios of Prepared Membranes

**Figure 2:**
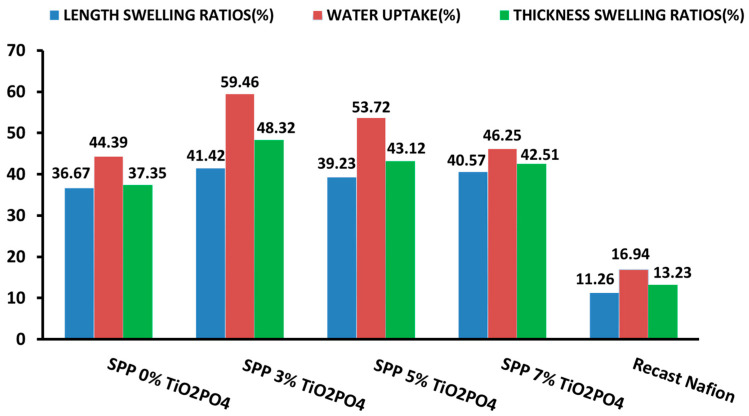
Water uptake and swelling ratio (in-plane and through-plane) at 80 °C.

Figure 2 shows the water uptake and swelling ratio of the membranes after soaking in deionized water at room temperature. First, the water uptake increased with increasing TiO_2_PO_4_ content, while the swelling ratio in all directions decreased; then, above 3% TiO_2_PO_4_, the trends were just reversed. The optimal level of TiO_2_PO_4_ is at 3%. The hydrophilicity of TiO_2_PO_4_ particles may be responsible for improved water uptake at low nanofiller content. In addition, the presence of TiO_2_PO_4_ particles may also increase the porosity of the membranes, which allows water molecules to pass through the membrane more easily, resulting in a higher water transport efficiency and, consequently, an increase in the water uptake of the synthesized membrane [41]. With larger amounts of TiO_2_PO_4_, other factors, such as those affecting crystallinity or microporous structure, may prevail and overcompensate the hydrophilic effect, so the combined effect of TiO_2_PO_4_ on water uptake and swelling degree may change. Comparing the result with those measured on recast Nafion, it is clearly observed that our blend membranes perform better than recast Nafion in terms of water uptake, while they are inferior in terms of swelling ratio. Unfortunately, the in-plane swelling of our membranes is 3–4 times larger. The in-plane swelling ratio should be kept low to moderate, as in-plane swelling can lead to mechanical damage of the membrane electrode assembly. In contrast, the through-plane swelling is beneficial, helping to establish proper contact between the MEA and the bipolar plates.

The water uptake ratios are very promising, suggesting that our membranes may have good water retention capacity, even under dry conditions. Consequently, composite membranes containing 3% TiO_2_PO_4_ may be advantageous for the operation of PEMFCs in applications where the PEM humidification is not easily manageable and, therefore, the PEM may be exposed to dry conditions.

### 3.2. Ion Exchange Capacity (IEC) of Prepared Membranes

**Figure 3:**
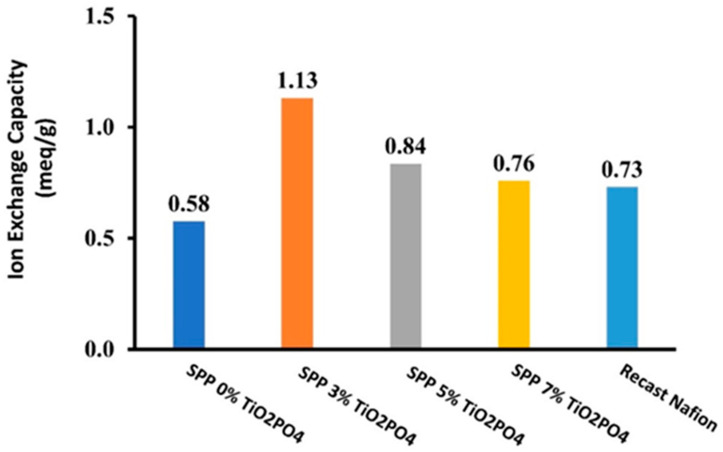
Ion exchange capacity for SPP blend membranes and recast Nafion.

Figure 3 shows the IEC results of the different blended membranes, and it can be seen that as TiO_2_PO_4_ content increases, the IEC passes through a maximum in the same way as in the case of water uptake.

The membrane containing 3% TiO_2_PO_4_ exhibited the highest IEC at 1.13 meq/g, whereas the parent SPP membrane showed an IEC of 0.58 meq/g. The presence of phosphate groups not only makes the membrane more hydrophilic but also means extra ion exchange sites. Increased water content and high IEC simultaneously within the membrane are critical, as they together provide a favorable environment for ion conduction [37]. The results of Figure 3 clearly prove that in the ion exchange capacity test, the synthesized membranes performed more efficiently than the recast Nafion. Therefore, the use of SPP membranes synthesized with 3% TiO_2_PO_4_ filler in a fuel cell should presumably lead to good results.

### 3.3. Thermogravimetric Analysis (TGA)

**Figure 4:**
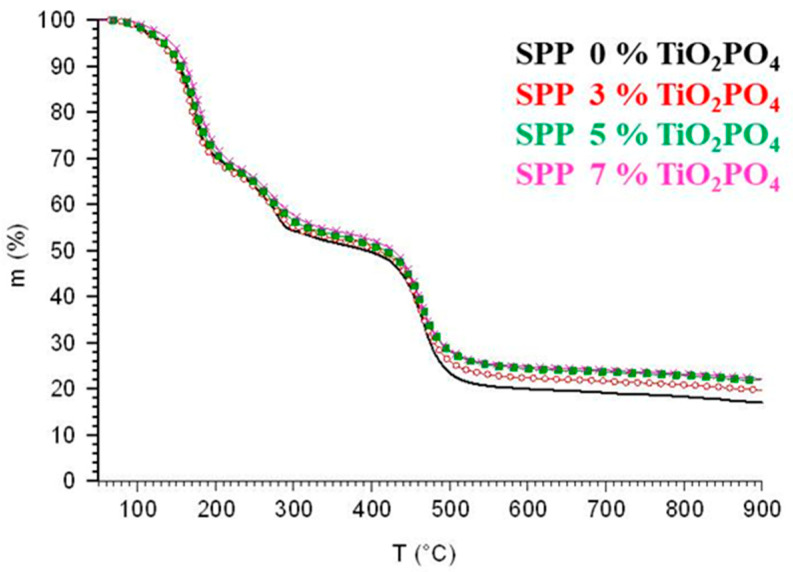
Thermogravimetric analysis for SSP blend membranes.

The thermal stability of SPP membranes was evaluated using thermogravimetric analysis, examining samples with different concentrations of TiO_2_PO_4_ under argon. Figure 4 shows the TGA results revealing three stages of weight loss. Each membrane first loses weight at around 175 °C, a loss of about 30%, probably due to moisture released from the membrane. After that, the disintegration of oxygen-rich functional groups in the composite membranes causes weight loss between 175 °C and 280 °C. Above 280 °C, further decomposition and structural breakdown of the membranes occur. Both the parent SPP membrane and the membranes loaded with the TiO_2_PO_4_ filler decompose according to similar patterns, with minor differences due to the amount of TiO_2_PO_4_ used. The presence of nanoparticles has no significant effect on the thermal stability and thermal degradation properties of the blend membranes. Table 2 contains information on the solid residue obtained when membranes were heated to 900 °C.

### 3.4. Scanning Electron Microscopy (SEM) Analysis

**Figure 5:**
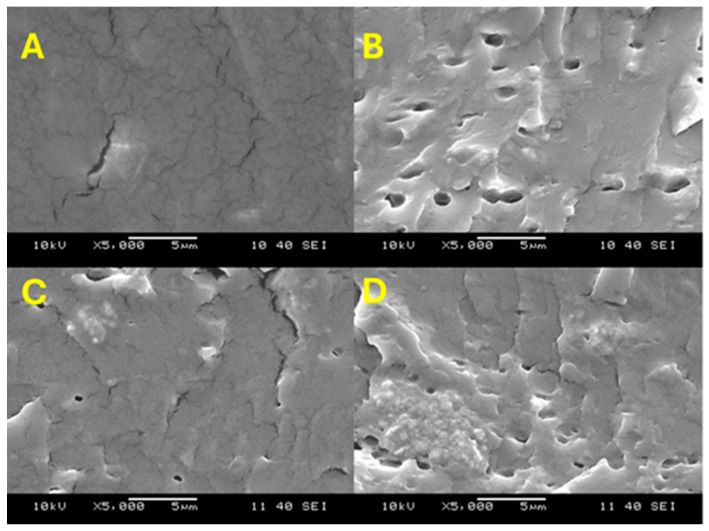
SEM images of cross-sections of SPP blend membranes (**A**, **B**, **C**, and **D** refer to 0%, 3% 5%, and 7% TiO_2_PO_4_, respectively).

**Figure 6:**
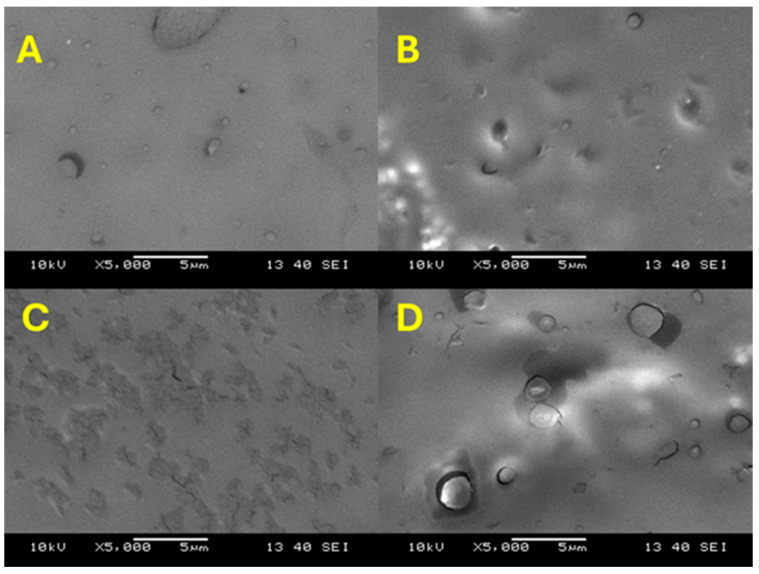
SEM images of the planar surface of SPP blend membranes (**A**, **B**, **C**, and **D** refer to 0%, 3%, 5%, and 7% TiO_2_PO_4_, respectively).

The surface morphology of the samples was analyzed using the scanning electron microscopy (SEM) method. Figure 5 and Figure 6 show the SEM images of SPP blend membranes. These micrographs demonstrate the compatibility and miscibility between the organic polymers and TiO_2_PO_4_. The uniform distribution of TiO_2_PO_4_ particles plays a crucial role in determining the thermal, mechanical, and electrochemical properties of the membranes. The images in Figure 5 show the distribution of the TiO_2_PO_4_ filler within the SPP matrix, while in Figure 6, we can see the planar distribution of the filler on the surface of membranes. Uniform distribution of the nanofiller can be seen. However, with too high a concentration of TiO_2_PO_4_, at 5% and 7%, the filler tends to accumulate and form spherical vesicles within the blended membranes, i.e., the uniform dispersion of the filler is lost.

### 3.5. Fourier Transform Infrared Spectroscopy (FTIR) Analysis

**Figure 7:**
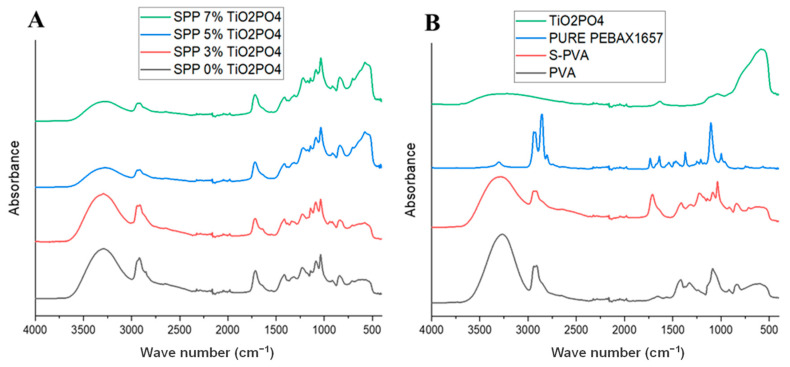
FTIR spectra of (**A**) SPP blend membranes and (**B**) the different constituents, such as titanium dioxide phosphate (TiO_2_PO_4_), PVA, and S-PVA, as well as the pure PEBAX 1657.

Fourier Transform Infrared (FTIR) Spectroscopy was employed to identify the functional groups and examine the interactions within SPP membranes between TiO_2_PO_4_ and organic constituents. Figure 7A shows the FTIR spectra. In the spectrum for the parent SPP membrane without the TiO_2_PO_4_ filler (shown by the black line), the bands can be attributed to the stretching vibrations for the PVA-based membranes. Characteristic absorption bands were observed for the C-O-C groups at 1020 cm^−1^, C-C bonds at 1130 cm^−1^, and ester carbonyl groups (-C=O) at 1730–1735 cm^−1^, consistent with esterification crosslinking between the hydroxyl groups in PVA and carboxyl groups in SSA. These features, highlighted in our previous article [38], confirm the chemical modifications introduced by SSA, as reflected in the spectrum in Figure 7B. The PEBAX-based membranes, on the other hand, displayed hydrogen bonding interactions between hydroxyl (OH) groups in PVA and carbonyl (C=O) groups in the amide segments of PEBAX. Figure 7A also illustrates the slight change in the spectrum of titanium dioxide phosphate (TiO_2_PO_4_) due to the gradual increase in filler ratios. The presence of TiO_2_PO_4_ was confirmed by the increase in intensity of the peak appearing at 630 cm^−1^, which was attributed to the vibrations of the Ti-O [37,42], and clearly shows the presence of the filler. Additionally, the bands observed at 2940 cm^−1^ and 3250 cm^−1^ are attributed to the stretching vibrations of the C-H and O-H groups, the intensity of which decreased with increasing the amount of TiO_2_PO_4_ filler compared to other parts of the PVA spectrum. The phenomenon indicates a strong contact between the filler and PVA, which may be more pronounced than the contact between the filler and PEBAX. Figure 7B presents the spectrum of TiO_2_PO_4_ particles; the band at 690 cm^−1^ corresponds to the stretching of the Ti-O bond [42]. The bands at 1028 and 1115 cm^−^^1^ are referred to as P-O stretching vibrations [43]. Additionally, Figure 7B illustrates the influence of the sulfonated process by SSA on the PVA spectrum; two distinctive peaks at 1101 cm^−1^ and 1036 cm^−1^ were identified, corresponding to the O=SO deformation vibration bands from SSA and confirming the presence of the SO_3_H functional group [24,38]. The peak observed at 1126 cm^−1^ is attributed to the deformation vibration of the C-O-C ester bonds. Table 3 illustrates all the FTIR peak assignments for SPP membranes and TiO_2_PO_4_ nanoparticles.

### 3.6. X-Ray Diffraction (XRD) Analysis

**Figure 8:**
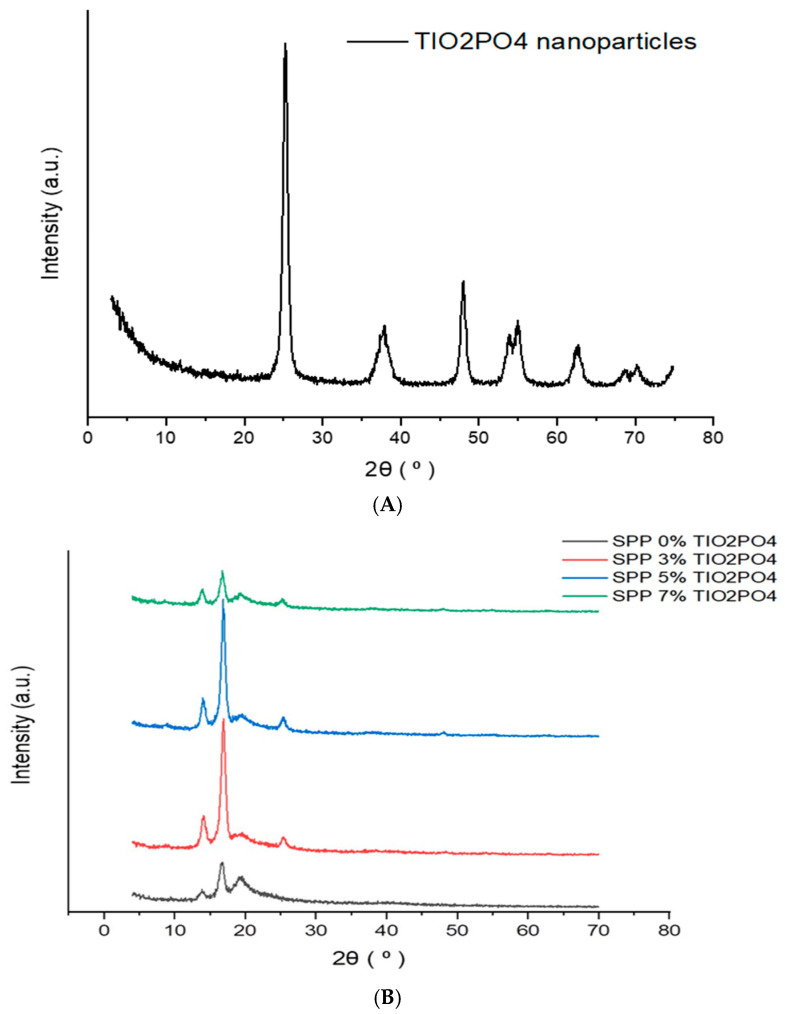
(**A**) X-ray diffraction for TiO2PO4 nanoparticles; (**B**) X-ray diffraction for SPP blend membranes.

X-ray diffraction was employed to identify the presence of crystallinity in various SPP membranes containing different amounts of TiO_2_PO_4_. Figure 8A shows the XRD pattern of TiO_2_PO_4_ nanoparticles. The analysis confirmed that the nanoparticles possess a crystalline anatase TiO_2_ structure. The crystallite size was estimated to be ~19 nm, while larger agglomerates of around 10 µm were also observed, indicating a particulate morphology. The material consists entirely of the anatase phase, with random orientation and good structural stability. The X-ray spectra shown in Figure 8B reveal the semicrystalline nature of the blend membranes. Four different peaks are observed at 2θ angles of approximately 14.2°, 17.3°, 20°, and 25°. PEBAX is a copolymer that consists of both crystalline and amorphous phases of polyamide (PA) and polyethylene oxide (PEO). The peaks observed at 14.2° and 17.3° might indicate a random arrangement of amorphous PEO and crystalline PA segments [31]. In addition, PVA is also a polymer with a partially crystalline structure. As mentioned in reference [44], the intermolecular hydrogen bonding between the PVA chains is responsible for the peak seen at 20°. Interestingly, the intensity of this peak decreases upon the addition of TiO_2_PO_4_, which may indicate that the presence of the nanofillers may reduce the number of intermolecular hydrogen bonds. In addition, a new peak can be detected at around the angle of 2θ ≈ 25° when the TiO_2_PO_4_ filler is present. Table 4 illustrates XRD peaks and their assignments for TiO_2_PO_4_ nanoparticles and proves the semicrystalline nature of the polymers that participate in blend membranes. According to our previous observation, this peak appears when a higher percentage of SSA molecules is added to the PVA chains. Similar to S-MMT, the TiO_2_PO_4_ filler enhances the crystallinity and makes the new phase visible by XRD, which can be due to SSA. Therefore, the primary effect of TiO_2_PO_4_ on sulfated PVA is an increase in crystallinity, which may also be accompanied by an attenuation of C-H and O-H stretching vibrations in the FTIR spectrum, as shown in Figure 7A. The better overall crystallinity achieved by the addition of the nanofillers is also indicated by the increase in the sharpness of the peak at 2θ ≈ 17.3°.

### 3.7. Mechanical Properties

**Figure 9:**
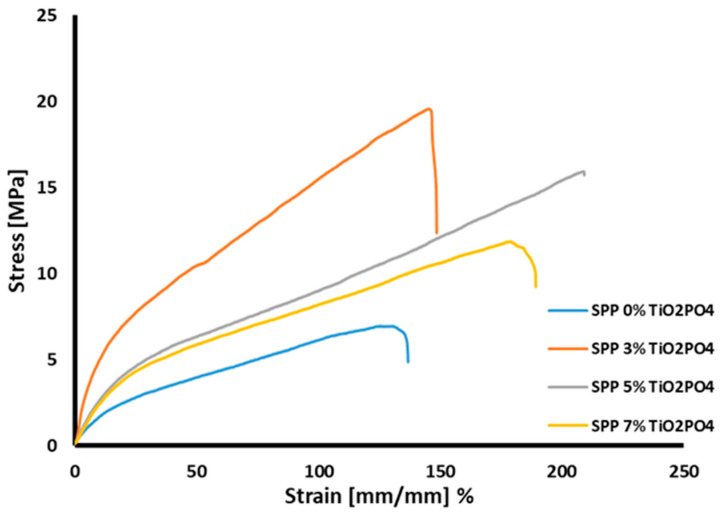
Mechanical stability for SSP blend membranes.

The mechanical strength of membranes is a crucial characteristic. It has been shown that the addition of small amounts of fillers to hybrid membranes can be an effective way to improve the mechanical properties of polymers [33]. Tensile tests were performed to evaluate the effect of TiO_2_PO_4_ nanofiller on the mechanical properties of SPP membranes. The results obtained on SPP membranes are shown in Figure 9. The results indicate that the parent blend membrane showed the lowest performance, with a maximum tensile strength of 5.8 MPa. The presence of TiO_2_PO_4_ nanofiller at concentrations of 5% and 7% led to an increase in membrane robustness, with a strength of 15.2 and 12.3 MPa and significant elongation at break. An even more pronounced improvement was achieved at 3% TiO_2_PO_4_ content, which led to an increased elongation at break and a tensile stress of 19.5 MPa. It is assumed that the uniform, fine distribution of TiO_2_PO_4_ nanoparticles is responsible for the better mechanical properties. This phenomenon is due to the fact that nanoparticles promote the distribution of energy in the material [42,45]. In addition, hydrogen bonds between the organic components and TiO_2_PO_4_ facilitate the dissipation of mechanical stresses. The composite membrane containing 3% TiO_2_PO_4_ may be a promising for PEMFC applications.

### 3.8. Chemical Stability Test

**Figure 10:**
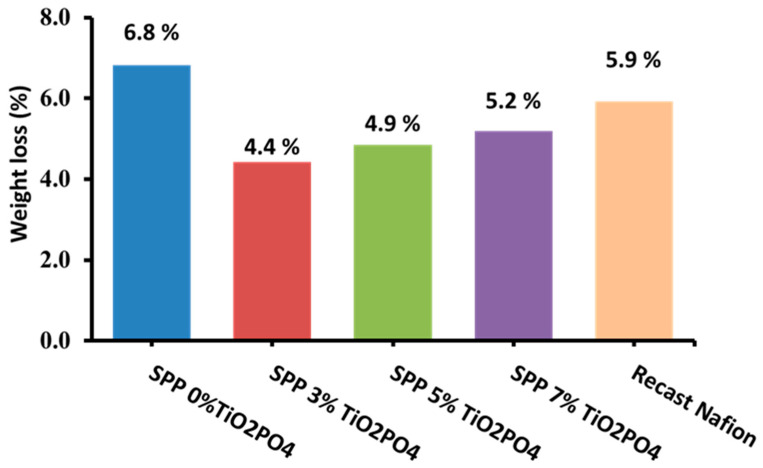
Chemical stability by Fenton’s test for SSP blend membranes.

Fenton’s reagent test, a common method for assessing chemical stability, is employed to determine the resilience of proton exchange membranes (PEMs) against radical species (OH· and OOH·) generated at the cathode in a PEM fuel cell. Figure 10 shows the percentage of mass loss of the produced membranes after exposure to Fenton’s reagent test. The membranes were immersed in the reagent for 24 h at 80 °C [38]. In the presence of the filler, the rate of weight loss decreased. This may be because TiO_2_PO_4_ has antioxidant or free radical-scavenging properties and can thereby reduce the effects of hydroxyl radicals [42,43]. This behavior can reduce the oxidative damage of the polymer matrix. In addition, hydrogen bonds and electrostatic interactions between TiO_2_PO_4_ and the hydroxyl groups of S-PVA and PEBAX 1657 promote the interfacial adhesion process, which also acts as a barrier against the oxidative behavior of Fenton’s reagent. The results shown in Figure 10 clearly proved that the chemical stability of the synthesized membranes was better than that of recast Nafion. The chemical stability of the synthesized membranes with 3% TiO_2_PO_4_ filler content was superior to that of other blended membranes. Therefore, we expect the synthesized SSP with 3% TiO_2_PO_4_ filler to perform well in the fuel cell.

### 3.9. Fuel Cell Performance Test

**Figure 11:**
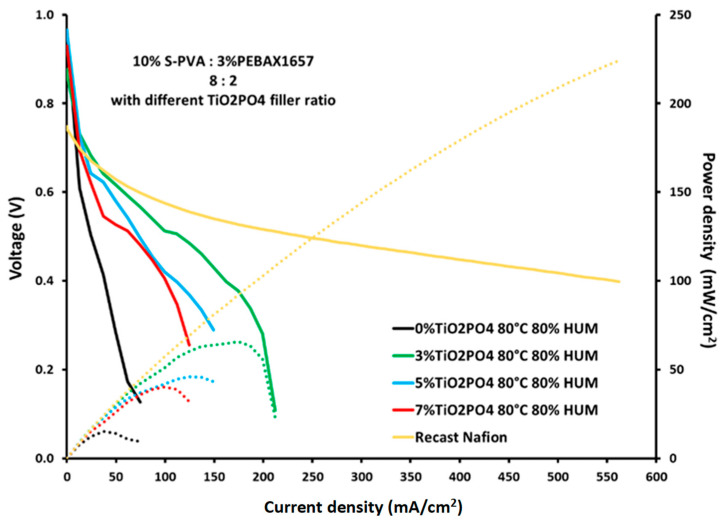
Polarization curves and power density curves for SPP blend membranes.

The electrochemical performance of membrane electrode assemblies, prepared with my blend membranes, was evaluated. The performance tests of membranes were performed using a single cell under controlled conditions at 80 °C and 80% humidity for both H_2_ and O_2_. Figure 11 presents the polarization and power density curves. The results show that the SPP membrane with 3% TiO_2_PO_4_ has the best current and power density of 175.5 mA/cm^2^ and 61.52 mW/cm^2^, respectively. It is the best membrane for fuel cell (FC) applications among all SPP membranes tested. The agreement between the ion exchange capacity (IEC) results, water uptake measurements, and SEM images predicted this excellent performance. Fine distribution of the nanofiller, good compatibility and miscibility of constituents, hydrophilicity, and the presence of proton exchange sites in high concentration proved to be a prerequisite to obtain a good membrane.

## 4. Conclusions

Titanium dioxide phosphate (TiO_2_PO_4_) nanoparticles were integrated into sulfonated polyvinyl alcohol (S-PVA) and PEBAX 1657 blend membranes. The functionalization of TiO_2_ with phosphate (PO_4_^3−^) anions was accomplished using an impregnation–calcination technique, ensuring stable chemical modification and improved compatibility with the polymer matrix. The controlled deposition of phosphate groups onto the TiO_2_ surface significantly enhanced the filler’s hydrophilicity and proton-conducting properties, making it an ideal additive for improving membrane performance.

The well-dispersed nanoparticles, whose homogeneous distribution within the SPP matrix was aided by continuous stirring and controlled solvent evaporation, created additional proton conduction channels while reinforcing the structural stability of the membrane. Not only the uniform distribution of the nanofiller, but also its good compatibility with S-PVA in particular, was confirmed by FTIR spectra and XRD patterns. The close contact between TiO_2_PO_4_ nanoparticles and S-PVA was evidenced by the change in the intensity of C-H and O-H stretching vibrations upon addition of the nanofiller. As nucleation centers, nanoparticles in proximity to S-PVA can induce crystallization of S-PVA, as confirmed by X-ray diffraction patterns. Eventually, it can be suggested that new conduction channels have formed within the PEBAX matrix, which contain S-SPA and TiO_2_PO_4_, and in which the interaction between phosphate groups and sulfonic acid groups is responsible for proton conduction.

The incorporation of 3 wt% TiO_2_PO_4_ nanoparticles into the SPP matrix resulted in significant improvements in different properties. The presence of TiO_2_PO_4_ improved water uptake and thermal resistance, which are key factors in determining membrane efficiency in fuel cell applications.

The ion exchange capacity (IEC) for the membrane containing 3 wt% TiO_2_PO_4_ showed the highest value at 1.13 meq/g, whereas the parent SPP membrane achieved an IEC of 0.58 meq/g. Among the tested compositions, the membrane with 3 wt% TiO_2_PO_4_ exhibited the highest electrochemical performance, reaching a peak current density of 175.5 mA/cm^2^ and a maximum power density of 61.52 mW/cm^2^.

Integration of nanofiller into the polymer matrix has a crucial role in improving the performance of non-fluorinated, environmentally friendly proton exchange membranes. The optimized combination of S-PVA, PEBAX 1657, and TiO_2_PO_4_ fillers offers a promising alternative to conventional fluorinated membranes, in line with sustainable and high-performance energy conversion technologies.

The findings of this study indicate that TiO_2_PO_4_-doped SPP membranes exhibit suitable structural, physicochemical, and electrochemical properties that make them promising membranes for practical use. Considering their enhanced stability and performance, the membranes can be commercialized in proton exchange membrane fuel cells (PEMFCs), which are highly renowned for portable and stationary power generation systems. Water management is a critical issue, for example, in the open cathode configuration with passive air breathing. Our membrane, which has significantly higher water uptake than recast Nafion, could provide a promising competitive advantage in these PEMFC stacks.

Further work could include, in particular, optimizing the nanofiller content, testing the long-term durability of the membranes under real operating conditions, and performing proton conductivity measurements to complement fuel cell performance tests. These directions could provide valuable information and advance the development of non-fluorinated composite membranes for fuel cell applications.

## Figures and Tables

**Table 1:** The designation of membranes.

Composition of Membranes	Name
S-PVA 8:2 PEBAX1657 without (TiO_2_PO_4_) filler	SPP 0% TiO_2_PO_4_
S-PVA 8:2 PEBAX1657 with 3% (TiO_2_PO_4_) filler	SPP 3% TiO_2_PO_4_
S-PVA 8:2 PEBAX1657 with 5% (TiO_2_PO_4_) filler	SPP 5% TiO_2_PO_4_
S-PVA 8:2 PEBAX1657 with 7% (TiO_2_PO_4_) filler	SPP 7% TiO_2_PO_4_

**Table 2:** Residues (%) after thermogravimetric analysis up to 900 °C.

Sample	Residue (%)
S-PVA—PEBAX 0% TiO_2_PO_4_	17.00
S-PVA—PEBAX 3% TiO_2_PO_4_	19.72
S-PVA—PEBAX 5% TiO_2_PO_4_	21.82
S-PVA—PEBAX 7% TiO_2_PO_4_	22.23

**Table 3:** FTIR peak assignments for SPP membranes and TiO_2_PO_4_ nanoparticles.

Wavenumber (cm^−1^)	Assignment	Origin/Interpretation
630	Ti–O stretching vibration	Confirms presence of TiO_2_PO_4_ filler
690	Ti–O stretching vibration	TiO_2_PO_4_ particles
1020	C–O–C stretching	PVA-based membranes
1028, 1115	P–O stretching	TiO_2_PO_4_ particles
1036, 1101	O=SO deformation vibrations	From SSA, confirming the SO_3_H functional group
1126	C–O–C ester deformation	PVA after sulfonation
1130	C–C stretching	PVA-based membranes
1730–1735	C=O stretching (ester carbonyl)	Esterification between PVA hydroxyl and SSA carboxyl groups
2940	C–H stretching	PVA/PEBAX membranes; intensity decreases with TiO_2_PO_4_ addition
3250	O–H stretching	Hydrogen bonding; intensity decreases with TiO_2_PO_4_ addition

**Table 4:** XRD peaks and their assignments for TiO_2_PO_4_ nanoparticles and SPP blend membranes.

2θ (°)	Assignment	Origin
~14.2	Crystalline PA segment	From polyamide domains in PEBAX copolymer
~17.3	Amorphous PEO chain arrangement	From polyethylene oxide domains in PEBAX
~20.0	Intermolecular hydrogen bonding in PVA	Partially crystalline structure of PVA; peak decreases upon addition of TiO_2_PO_4_ due to reduced hydrogen bonding
~25.0	TiO_2_PO_4_ nanofiller peak	New peak appearing with TiO_2_PO_4_ incorporation, confirming filler presence

## Data Availability

The data presented in this study are available upon request from the corresponding author. The data are not publicly available due to their unstandardizable complexity.
